# The futility of long-term predictions in bipolar disorder: mood fluctuations are the result of deterministic chaotic processes

**DOI:** 10.1186/s40345-021-00235-3

**Published:** 2021-10-01

**Authors:** Abigail Ortiz, Kamil Bradler, Maxine Mowete, Stephane MacLean, Julie Garnham, Claire Slaney, Benoit H. Mulsant, Martin Alda

**Affiliations:** 1 Department of Psychiatry, University of Toronto, Toronto, ON Canada; 2 Centre for Addiction & Mental Health, CAMH 100 Stokes St., Rm 4229, Toronto, ON M6J 1H4 Canada; 3 ORCA Quantum Computing, Toronto, ON Canada; 4 Department of Electrical Engineering, University of Ottawa, Ottawa, ON Canada; 5 Institute for Mental Health Research, The Royal Ottawa Hospital, Ottawa, ON Canada; 6 Nova Scotia Health Authority, Halifax, NS Canada; 7 Department of Psychiatry, Dalhousie University, Halifax, NS Canada; 8 National Institute of Mental Health, Klecany, Czech Republic

**Keywords:** Bipolar disorder, Mood fluctuations, Nonlinear analyses, Episode prediction, Unaffected first-degree relatives

## Abstract

**Background:**

Understanding the underlying architecture of mood regulation in bipolar disorder (BD) is important, as we are starting to conceptualize BD as a more complex disorder than one of recurring manic or depressive episodes. Nonlinear techniques are employed to understand and model the behavior of complex systems. Our aim was to assess the underlying nonlinear properties that account for mood and energy fluctuations in patients with BD; and to compare whether these processes were different in healthy controls (HC) and unaffected first-degree relatives (FDR). We used three different nonlinear techniques: Lyapunov exponent, detrended fluctuation analysis and fractal dimension to assess the underlying behavior of mood and energy fluctuations in all groups; and subsequently to assess whether these arise from different processes in each of these groups.

**Results:**

There was a positive, short-term autocorrelation for both mood and energy series in all three groups. In the mood series, the largest Lyapunov exponent was found in HC (1.84), compared to BD (1.63) and FDR (1.71) groups [F (2, 87) = 8.42, p < 0.005]. A post-hoc Tukey test showed that Lyapunov exponent in HC was significantly higher than both the BD (p = 0.003) and FDR groups (p = 0.03). Similarly, in the energy series, the largest Lyapunov exponent was found in HC (1.85), compared to BD (1.76) and FDR (1.67) [F (2, 87) = 11.02; p < 0.005]. There were no significant differences between groups for the detrended fluctuation analysis or fractal dimension.

**Conclusions:**

The underlying nature of mood variability is in keeping with that of a chaotic system, which means that fluctuations are generated by deterministic nonlinear process(es) in HC, BD, and FDR. The value of this complex modeling lies in analyzing the nature of the processes involved in mood regulation. It also suggests that the window for episode prediction in BD will be inevitably short.

**Supplementary Information:**

The online version contains supplementary material available at 10.1186/s40345-021-00235-3.

## Background

Bipolar disorder (BD) is a mood disorder characterized by (hypo)manic, mixed, or depressive episodes, interspersed with low amplitude mood fluctuations or, at times, with subsyndromal mood symptoms. Understanding how mood is regulated is important in BD, as we are only starting to conceptualize it as a more complex disorder than simply one of recurring manic and depressive episodes.

Mood regulation is a complex and poorly understood process, which can be conceived as a “buffer” system that allows flexible responses to changing conditions (Ortiz and Alda 2018). All complex systems, including mood, are continuously subject to stochastic variations in external conditions. From a dynamic point of view, the properties that enable the system to adapt to these stochastic variations are considered “complex”. These properties include: (i) nonlinearity, i.e., systems do not respond in a way that is proportional to the amount they are stimulated; (ii) the lack of a single or characteristic scale, i.e., fractal organization; and (iii) emergent properties, i.e., properties that emerge from the whole but were not present in the parts. Nonlinearity, however, does not imply a lack of underlying structure; on the contrary, nonlinear systems have organized, discoverable principles. The challenge is that these principles are difficult to uncover by conventional analyses and require nonlinear methods for their study.

Nonlinear methods offer new tools to quantify, model, and predict the behavior of complex systems (Ehlers 1995; Pincus 2001). Time-series analysis is a type of nonlinear method employed to study a collection of observations made sequentially in time (Chatfield 2016). Its special feature is that successive observations are not independent, and that the analysis must take into account the time order of the observations. Using time-series analysis, we have recently described higher regularity—a “pathologically stable” or organized mood in patients with BD (Ortiz et al. 2015) and their unaffected first-degree relatives (FDR) (Ortiz et al. 2019). Other authors have also described that mood in patients with BD is a process that becomes more organized over a longer time scale, as opposed to processes in healthy controls (Gottschalk et al. 1995; Bayani et al. 2017; Bonsall et al. 2012).

Although this increased degree of organization of mood in BD may seem paradoxical, these data fit an increasingly apparent pattern in Biology, where aging (Goldberger et al. 2002a, b), cardiovascular diseases (Jelinek et al. 2013; Olde Rikkert et al. 2016; Peng et al. 1995a), metabolic diseases (Wu et al. 2013; Gomolka et al. 2018), and psychiatric diseases (Cowdry et al. 1991; Golier et al. 2001; Katerndahl et al. 2007; Pincus et al. 2008) are marked by increasing degrees of organization. In other words, these systems are less flexible and able to cope with the demands of a constantly changing environment. These observations have led to hypothesize that mood variability might be better described in terms of chaotic dynamics (Bonsall et al. 2012; Bonsall et al. 2015; Huber et al. 1999, 2000).

In this paper, we aim to further characterize the nonlinear dynamics of mood and energy regulation in time-series data from 90 participants (30 healthy controls (HC), 30 euthymic patients with BD, and 30 unaffected FDR) using nonlinear methods. While our research shares a foundation with our previous work using paper-based time-series analysis to compare mood regulation in HC, unaffected FDR, and euthymic patients with BD, in this study we analyzed mood and energy series using three nonlinear indicators (Lyapunov exponent, detendred fluctuation analysis, and fractal dimension), with the aim of understanding whether mood fluctuations stem from different processes in each of these groups.

## Material and methods

### Participant recruitment

As per our previous papers (Ortiz et al. 2015, 2019), we obtained data from 90 participants: 30 HC, 30 euthymic patients with BD, and 30 unaffected FDR. Euthymia was operationalized as at least 3 months of a Young Mania Rating Scale (YMRS) score ≤ 5 (Young et al. 1978) and a Hamilton Depression Rating Scale (HDRS) score ≤ 7 (Hamilton 1960). All participants were interviewed by the same investigator (AO), using the Schedule for Affective Disorders and Schizophrenia-Lifetime version (SADS-L) (Endicott and Spitzer 1978); diagnosis was confirmed in a blind fashion in consensus meetings with the research team. The investigation was carried out in accordance with the latest version of the Declaration of Helsinki. The study design was approved by the local ethical committee and written informed consent of participants was obtained after the nature of the procedures had been fully explained.

### Measurements

We used paper-based visual analog scales (VAS) to measure mood, energy, anxiety, and sleep. The scale ranges from ‘1’ to ‘9’, with ‘5’ being ‘their usual’. Participants provided measurements on these variables over a three-month period, twice each day. The first rating was completed 1 h after waking up, and the second rating 1 h before bedtime. For incomplete data in the VAS scale (2 days in a row), we used interpolation methods; if more than 2 days were missing consecutively (usually at the end of the study), the rest of the data were removed from the calculations.

### Analytical consideration for nonlinear analyses

1. Time-series analyses
  The analyses performed relies on three assumptions:
  (i) The character of the collected data: the datapoints *Si (*for *i* = *{1, 2,9}*), represent an interval scale. This means that we consider the data not only to be a totally ordered set *{Si} (S*_*i*_ < *S*_*i*+*1*_ for all measured quantities) but also that the differences between *i* and *j* (*i ≠ j*) do have a specific meaning. This is a strong assumption, given that the underlying process(es) we try to shed light on need not be proportional to the measurement scale and most likely are logarithmic, as seen in other human phenomena (Varshney and Sun 2013).
  (ii) The validity of a model to which we try to fit the data. The collected data are noisy, and the resolution in both the domain and range of the time series is low. We were cautious not to overfit the data by using simple models (i.e., first-order departure from a completely random process (white noise) or the autoregressive process AR (Ortiz and Alda 2018).
  (iii) Finally, given the character of the data, we cannot verify the requirement of strict stationarity, but we may relax this notion and ask the time series to satisfy *weak* stationarity. That is, we require only the first two moments (mean and variance) to be time invariant. The requirement of at least weak stationarity is crucial for time-series analysis. For a complete description of the analyses, please see (Ortiz et al. 2015).
  In summary, we assumed that the difference between intervals in the scale has a specific meaning and that the time series are weak non-stationary. We were cautious not to overfit the data by using simple models.
2. Lyapunov exponent (LE)
  LE is a quantitative measurement of a parameter’s sensitive dependence on initial conditions, which provides a quantitative indication of the chaotic level of a system. In medicine, LE has been used to analyze heart rate variability (Hu et al. 2009 and EEG signals (Hu et al. 2009; Hu et al. 2010). LE defines the average rate of divergence or convergence of two neighboring trajectories in the state-space (Nayak et al. 2018). Positive LE values indicate that the phase space trajectories are diverging (i.e., the closely located points in the initial state are quickly separating from each other in the *i* direction), and the system is losing its predictability, exhibiting chaotic behavior. Conversely, a negative LE represents the average rate of the convergence of the phase space trajectories. Additional file 1 describes specific analytical considerations for LE.
3. Detendred fluctuation analysis (DFA)
  DFA is used to measure long range dependence in a time-series (Peng et al.  1994, 1995b). A parameter referred as “Hurst exponent” (*H*) describes the correlation properties of the dataset; it quantifies the relative tendency of a time series cluster in a direction over time. A value *H* in the range 0.5–1, indicates a time series with positive autocorrelation, which means that a high value in the series will probably be followed by another high value. A value in the range of 0–0.5 indicates a time-series with long-term switching between high and low values in adjacent pairs, which means that a single high value will be probably followed by a low value and that the value after that will tend to be high, with this tendency to switch between values lasting a long time into the future. Additional file 1 describes specific analytical considerations for DFA.
4. Fractal dimension (*D*)
  *D* is a ratio providing a statistical index of complexity, and a highly sensitive measure for detecting hidden information contained in physiological time series (Raghavendra and Dutt 2010). There are many methods used to calculate *D*, but the most accurate estimate is achieved by Higuchi’s method. Higuchi’s fractal dimension originates from chaos theory and for almost 30 years it has been successfully applied as a complexity measure of artificial or physiological signals, including EEG and EKG (Gomolka et al. 2018; Skinner et al. 1992; Kesic and Spasic 2016; Ma et al. 2018). Values associated with natural phenomena are estimated to be between 1 and 2 (Higuchi 1988). A value of 1 corresponds to a regular time-series; while for Gaussian-type noise, it might attain higher values; i.e., 1.5 for Brownian, 1.8 for pink noise, and 2.0 for white noise (Gomolka et al. 2018). For information on the calculation of *D*, please refer to Additional file 1.

### Statistical analyses

After all nonlinear parameters had been calculated, we conducted analyses of variance for each of them (LE, DFA, and *D*) to determine whether there were any differences between the three groups in mood or energy series. All analyses were performed using MATLAB ®.

## Results

### Demographic and clinical characteristics of the sample

Ninety participants were included in the analyses: 30 HC, 30 euthymic BD and 30 FDR. In the BD subgroup, 76.6% were BD type I and all patients were on long-term pharmacotherapy (see Table 1). With proportions of participants completing >84 days of consecutive entries of 96% in the HC group, 93.3% in the BD group, and 86.6% in the FDR groups, we analyzed 14,980 datapoints (5200 in the HC group, 4970 in the BD group and 4810 in the FDR group). Please refer to our original paper for the complete description of the sample and distribution of individual measures (Ortiz et al. 2019).

**Table 1:** Pharmacotherapy (patients with BD, N = 30)

Medication	% of patients
Mood stabilizers	90
Lithium carbonate	50
Lamotrigine	20
Valproic acid	13.3
Carbamazepine	6.7
None	10
Combination treatment with antidepressants	30
SSRI	3.3
SNRI	3.3
MAOI	13.3
Other	10
None	70
Combination treatment with antipsychotics	50
First-generation	6.7
Second-generation	43.3
None	50
Other treatments	43.2
Sleep-inductors/benzodiazepines	16.6
Thyroid supplementation	20
Tryptophan	3.3
Atomoxetine	3.3
None	56.8

MAOI: monoamine oxidase inhibitor; SNRI: serotonin–norepinephrine reuptake inhibitor; SSRI: selective serotonin reuptake inhibitor

#### Lyapunov exponent (LE)

Table 2 describes LE for all groups, for both mood and energy series. For the mood series, the largest LE was found in HC (1.84), compared to BD (1.63) and FDR (1.71). An ANOVA yielded a statistically significant difference between groups (F (2, 87) = 8.42, p < 0.005), with a post-hoc Tukey test showing that LE was significantly higher in HC than in the BD (p = 0.003) and FDR groups (p = 0.03). Similarly, for the energy series, HC showed a LE of 1.85, compared to 1.76 for BD and 1.61 for FDR (F (2, 87) = 11.02; p < 0.005), with a post-hoc Tukey test showing that LE was significantly higher in HC group compared to FDR group (p < 0.001), but not the BD group (p = 0.1).

**Table 2:** Nonlinear coefficients for mood and energy series

Coefficient	Series	HC (mean ± SD)	BD (mean ± SD)	FDR (mean ± SD)	Statistic
LE	Mood	1.84	1.63	1.71	F(2, 87) = 8.42; p < 0.005
	Energy	1.85	1.76	1.61	F(2, 87) = 11.02; p < 0.005
DFA	Mood	0.53 ± 0.03	0.50 ± 0.01	0.52 ± 0.03	F(2, 87) = 0.14, p = 0.86
	Energy	0.54 ± 0.04	0.54 ± 0.04	0.53 ± 0.03	F(2, 87) = 0.02; p = 0.97
Fractal Dimension	Mood	1.54 ± 0.11	1.51 ± 0.05	1.52 ± 0.04	F(2, 87) = 1.48, p = 0.2
	Energy	1.42 ± 0.12	1.41 ± 0.05	1.41 ± 0.06	F(2, 87) = 0.18, p = 0.8

LE: Lyapunov exponent; DFA: Detrended fluctuation analysis; HC: Healthy controls; BD: bipolar disorder; FDR: Unaffected first-degree relatives

#### Detrended fluctuation analysis (DFA)

Table 2 describes the Hurst (*H*) exponent for all groups, for both mood and energy series. There were no statistically significant differences between groups for either mood or energy series (p > 0.5).

#### Fractal dimension (*D*)

Table 2 describes *D* for all groups, for both mood and energy series. There were no statistically significant differences between groups for either mood or energy series (p > 0.05).

## Discussion

The underlying nature of mood variability is in keeping with that of a chaotic system, rather than noise, which means that fluctuations are generated by deterministic nonlinear process(es) in HC, BD, and FDR participants. The three different techniques used in these analyses have all demonstrated that chaotic behavior can originate from deterministic systems that have simple mathematical descriptions (Klonowski 2007).

Our main findings include a positive, short-term, autocorrelation in the time-series for mood and energy series in all groups; with a more chaotic pattern in the time-series for mood in HC, than in BD or FDR participants, which is in keeping with our previous results.

The limitations of this analysis include using one-dimensional time-series, as these may not not sufficient to reconstruct the complex dynamics of mood regulation. However, despite the ‘low resolution’ of the visual analog scale employed, this is a measure that has been validated in mood disorders and it meets the assumptions needed to validate the analysis employed. Moreover, using nonlinear methods allowed us to detect hidden information in physiological time-series, which supports conceptualizing mood variability as an inherent nonlinear process.

LE showed differences between the three groups: for both mood and energy series, LE was higher in HC than in BD and FDR participants, which is indicative of a more chaotic pattern. As mentioned above, LE is a nonlinear parameter that quantifies the sensitivity to initial conditions. The existence of a positive exponent for almost all initial conditions in a bounded dynamic system is a widely used definition of deterministic chaos (Zhong et al. 2007; Gao et al. 2007). Positive values (as shown in all groups) mean that two trajectories starting very close together will rapidly diverge from each other, and thereafter have totally different futures. The practical implication is that long-term prediction becomes impossible in such a system, because small uncertainties are quickly amplified. This is relevant when it comes to calculating the window of episode prediction: a positive LE indicates the futility of trying to predict the detailed long-term behavior of a chaotic system. However, short-term prediction may be feasible in BD, as: (i) mood can be considered as a short-term memory process (Ortiz et al. 2015); (ii) our previous studies using machine-learning models for episode prediction have shown the last 4 days as preferred over other windows of prediction (Ortiz et al. 2018).

For DFA, our results show that all three groups have *H* values close to 0.5, which is indicative of a positive autocorrelation in their time series; i.e., a high value in the series will probably be followed by another high value. This result is consistent with our prior finding of an ARIMA (1,1,0) model, that conceptualizes mood as a short-term memory process (Ortiz et al. 2015) and with prior studies that also described a lag of 1 day in mood fluctuations (Gottschalk et al. 1995; Werf et al. 2006). This process could explain the daily fluctuations (perturbations or “micro-recoveries”) in mood seen in all groups. This behavior will likely be different in untreated patients, patients with mood symptoms but not a primary mood disorder (e.g., borderline personality disorder) or in patients with BD experiencing an acute episode.

Lastly, in the fractal dimension analysis, all three groups have *D* values close to 1.5, which is in keeping with the values in time-series associated with natural phenomena. As a highly sensitive measure for detecting hidden information contained in physiological time series, our results show, as expected, an underlying complexity in mood variability in the three groups.

Overall, these findings suggest that a more chaotic pattern is present in healthy systems; whereas compromised systems show a more rigid pattern, not as flexible or resilient to adjust to the demands of a changing environment. Resilience is defined as the capacity to tolerate disturbance without collapsing (Clements and Ozgul 2018). Efforts to understand resilience in humans are relatively advanced in Geriatrics (Olde Rikkert et al. 2016; Lagro et al. 2012) and Cardiology (Quail et al. 2015). The breakdown of resilience proposed only recently as a characteristic of mood disorders (Ortiz and Alda 2018; Golier et al. 2001; Cochran et al. 2018; Scheffer et al. 2018; Kossakowski et al. 2019; Leemput et al. 2014). As resilience approaches zero, a critical transition can be triggered even by a minor event (Scheffer et al. 2001, 2009; Nelson et al. 2017; Nes et al. 2016; O'Regan and Burton 2018). In other words, slower recovery from small perturbations is an indicator that the system is in a fragile state and that a tipping point might be near (Nelson et al. 2017).

Our findings have several clinical implications. First, it is important to recognize that mood variability is an important characteristic of healthy systems, and we can inform clinicians and patients about the importance of these ‘low amplitude’ mood fluctuations. Patients do not need to feel ‘flat’ to be stable. Second, we should recognize the role of other physiological factors, such as sleep, in increasing the ability of the system to recover from small perturbations. Cognitive strategies could also be important tools to help decrease co-morbid anxiety or substance use disorders, which can easily be “the tipping point” for a system that is already fragile. By increasing the system’s resilience, it would be more prone to small, but not critical transitions (e.g., minor life events would lead to adjustment disorder symptoms, rather than a depressive episode).

Other future clinical applications from our findings include the use of time-series analysis to assess mood variability in difficult diagnostic cases (e.g., borderline personality disorder versus bipolar disorder). Finally, nonlinear techniques can be used to model clinical trajectories and forecast the onset of depressive, manic, or mixed episodes, keeping in mind that these will be necessarily short-term forecasts.

## Conclusions

Mood fluctuations are generated by deterministic chaotic process(es) in healthy controls, patients with BD and their unaffected first-degree relatives. Mood regulation is a short-term memory process, which implies that it is autocorrelated in the short term (e.g., mood today will be correlated with mood tomorrow, but not farther than that). This has clinical implications when predicting mood episodes in BD: due to the underlying nature of mood fluctuations, only short-term forecasts are possible.

With the uptake in passive sensing and smartphone data to improve clinical care in BD (Ortiz et al. 2021), including predicting episodes of illness, we need to be aware that mood is regulated by a system sensitive to initial conditions. This means that two trajectories starting very close together will rapidly diverge from each other, and thereafter have completely different futures. The practical implication is that long-term prediction becomes impossible in a system like this, in which small uncertainties are quickly amplified. In other words, it is futile trying to predict the detailed long-term behavior of a chaotic system. Instead, we should focus on demonstrating the feasibility and clinical impact of short-term prediction in BD.

In closing, living systems are complex and operate far from equilibrium. Mood disorders, like any other clinical disorder, should be conceptualized as the result of a loss of complexity, rather than the loss of regularity.

## Supplementary Information

**Additional file 1.** Analytical considerations and calculations for LE, DFA and *D*.

## Data Availability

Data sharing is not applicable to this article as no datasets were generated or analyzed during the current study.
