# Supplementary material for: The futility of long-term predictions in bipolar disorder: mood fluctuations are the result of deterministic chaotic processes
Source: Int J Bipolar Disord. 2021 Oct 1;9:30. doi: 10.1186/s40345-021-00235-3 (PMC8486895; doi:10.1186/s40345-021-00235-3)
Supplement: Supplementary file 1 — Additional file 1. Analytical considerations and calculations for LE, DFA and D. [file 40345_2021_235_MOESM1_ESM.docx]

**Additional file**

**Detrended Fluctuation Analysis (DFA)**

Following (55), the original (noisy) time series *{x_k_}, k = 1, 2, …, N* is first integrated to yield the signal

$$x\left( m \right)=\sum_{i=1}^{m} \left( x_{i}-\left\langle x \right\rangle\right),$$

(1)

where 1 ≤ *m* ≤ *N* and

__

(2)

represents the arithmetic mean of the time series. Next, the time series is divided into boxes of equal size (represented by N), and the integrated time series is fitted through the use of a polynomial function, *X_fit_(k),* conventionally referred to as the ‘local trend’ (55). For order-*l* DFA (DFA-1 if *l* = 1, DFA-2 if *l* = 2m, etc.), the *l*-order polynomial function should be applied for the fitting.

Subsequently, the integrated time series is detrended through a subtraction of the local trend in each box. This gives the detrended fluctuation function *X(k)* as

__

(3)

Finally, for each box of size N, the root-mean-square (rms) fluctuation is computed according to


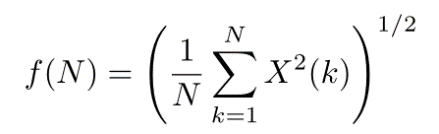


(4)

and a relationship between N and *f(N* *)* is thus developed. A power-law relationship of the type *_F(N)_ ~ n ^α^* implies the presence of scaling, and the parameter $\alpha$ (referred to as the ‘scaling exponent’ or ‘correlation exponent’ ) describes the correlation properties of the signal.

**Fractal Dimension**

Fractal dimension (D) is a statistical measure based on an algorithm (56) which gives an indication of how fully a particular element fills the space in which it is located. It corresponds to the self-similarity of the signal, i.e., the greater the fractal dimension value the more irregular the signal will be, indicating increased complexity. An effective technique for calculating fractal dimension is Higuchi’s algorithm (37, 56). Accordingly, given the finite data set $\left\{ y\left( 1 \right),y\left( 2 \right),\ldots,y\left( n \right) \right\}$, where, in this case, ‘n’ represents the number of mood samples, a new data set can be reconstructed, such that a Fractal Dimension can be determined as follows:

First, a time series of dimension k is formed according to

$$Y{}_{k}^{n}{}=\left\{ x\left[ n \right],x\left[ n+k \right],x\left[ n+2k \right],\cdots x\left[ n+int \left( \frac{N-n}{k} \right),k \right] \right\}$$

(5)

provided that *k* and *n* are integers, and that the function ‘*int(*)’* returns the integer part of its argument. Also, *k* represents the discrete time interval between data points, and *n = 1, 2, . . . , k* prescribes the initial time value (57). Next, the length of each new time series is computed as


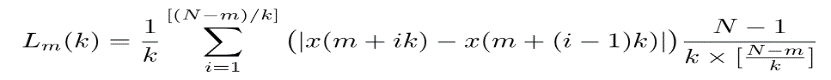


(6)

in which N remains the length of the original data set, and the term $\frac{N-1}{int \left( \frac{N-n}{k} \right)k}$ stands for a ‘normalization factor’. In the third step of the algorithm, the average of the *k* values $\left\{ L\left( n,k \right),n=1,2,\cdots,k \right\}$, is computed as


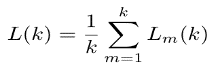


(7)

to define the curve’s length for the time interval represented by *k*. And in the final step, a log-log plot of

$$L\left( k \right)vs \frac{1}{k}, k=1, 2, \ldots, kmax$$

(8)

yields a straight line, whose slope is the fractal dimension (FD) of the of the time series *Y* constructed in step 2. Doyle (58) describes the maximum number $\left( k_{max}() \right)$of sub-series’ as the point at which the FD plateaus.

**Lyapunov Exponent**

The method adopted is that described by Rosenstein (59), in which an attractor dynamics is constructed from the mood samples data set, with the use of the delay method. Its starting point defines the reconstructed trajectory as the m-tuple X considered as a column vector, given as

*X = [X_1, …, X_M]*

(9)

with each X representing the system’s state at the discrete time denoted by *‘I*’. If there are ‘*N’* points in the time series, then,

__

(10)

Where ‘*j*’ denotes the reconstruction delay, and ‘*m’*, the embedding dimension of the trajectory. So that X of eqn. (9) becomes an M times m matrix, provided that

$M=N-\left( m-1 \right)j$

(11)

Rosenstein (59) describes that in the typical case, ‘*m*’ is estimated with the use of the Takens criterion, and prescribes a number of options for specifying the reconstruction delay. The dynamics reconstruction stage is followed by the location of the *nearest neighbor* for each point __ on the trajectory; and this done, for some particular reference point X_k_ by determining the point for which the Euclidean distance to X_k_ is minimized. That is determining

__

(12)

In which $d{}_{k}{}\left( 0 \right)$denotes the initial Euclidean distance from the k-th point to its nearest neighbour, and the function ||.|| returns the Euclidean norm of its argument. By imposing the additional constraint that the temporal separation of nearest neighbours should be greater than the mean period of the time series, the algorithm is enabled to consider each pair of nearest neighbours as initial conditions for different trajectories (59). Thereafter, the largest Lyapunov exponent is determined as the mean of the rate of the separation of nearest neighbours. In particular, the form utilized for this work is that given by (59) as


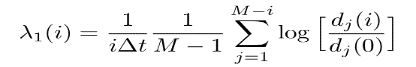


(13)

In eqn. (13), $\Delta t$represents the time series’ sampling period, and *d _j_ (i)* is the distance between the *j^th^* pair of nearest neighbours after *i* discrete time steps; i.e., $\left( i \cdot\Delta t \right)$.

The algorithm proceeds by assuming that the rate of divergence of the *j^th^* pair of nearest neighbours is approximately given by

*d_j_ (i) ≈ C_j_e*__ *^(i.∆t)^*

(14)

or, equivalently,

$log{}_{e}{} C_{j}+\lambda{}_{l}{}\left( i\Delta t \right)$

(15)

Equation (15) describes a set of approximately parallel lines, each with a slope of approximately__. As remarked by (59), eqn.(14) and eqn. (15), the normalization is not required for the computation of an estimate for __. For this reason, the largest Lyapunov exponent (particularly when small, noisy data sets of the type of interest here are being processed) is readily and accurately determined with the use of a least-squares curve fitting approach for the ‘average line’, defined by


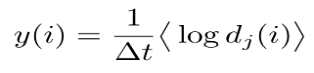


(16)

where ‹…› denotes the average values of *j*.
